# Comparative in vitro treatment of mesenchymal stromal cells with GDF-5 and R57A induces chondrogenic differentiation while limiting chondrogenic hypertrophy

**DOI:** 10.1186/s40634-023-00594-z

**Published:** 2023-03-21

**Authors:** Manuel Weißenberger, Mike Wagenbrenner, Joachim Nickel, Rasmus Ahlbrecht, Torsten Blunk, Andre F. Steinert, Fabian Gilbert

**Affiliations:** 1 Department of Orthopaedic Surgery, Center for Musculoskeletal Research, Julius-Maximilians-University Würzburg, König-Ludwig-Haus, Würzburg, Germany; 2 Department of Orthopedic Surgery, University of Wuerzburg, König-Ludwig-Haus, Brettreichstraße 11, 97074 Würzburg, Germany; 3 Department of Orthopaedics and Trauma Surgery, Musculoskeletal University Center Munich (MUM), University Hospital, LMU Munich, Marchioninistraße 15, 81377 Munich, Germany; 4 Department of Tissue Engineering and Regenerative Medicine, Julius-Maximilians-University Würzburg, University Hospital, Würzburg, Germany; 5 Department of Trauma-, Hand-, Plastic- and Reconstructive Surgery, Julius-Maximilians-University Würzburg, University Hospital, Würzburg, Germany; 6 Current address:, Department of Orthopaedic, Trauma, Shoulder and Arthroplasty Surgery, Rhön-Klinikum, Campus Bad Neustadt, Bad Neustadt, Germany

**Keywords:** Bone marrow, Cartilage, Chondrogenesis, Chondrogenic hypertrophy, Mesenchymal stromal cell, GDF-5, R57A

## Abstract

**Purpose:**

Hypertrophic cartilage is an important characteristic of osteoarthritis and can often be found in patients suffering from osteoarthritis. Although the exact pathomechanism remains poorly understood, hypertrophic de-differentiation of chondrocytes also poses a major challenge in the cell-based repair of hyaline cartilage using mesenchymal stromal cells (MSCs). While different members of the transforming growth factor beta (TGF-β) family have been shown to promote chondrogenesis in MSCs, the transition into a hypertrophic phenotype remains a problem. To further examine this topic we compared the effects of the transcription growth and differentiation factor 5 (GDF-5) and the mutant R57A on in vitro chondrogenesis in MSCs.

**Methods:**

Bone marrow-derived MSCs (BMSCs) were placed in pellet culture and in-cubated in chondrogenic differentiation medium containing R57A, GDF-5 and TGF-ß1 for 21 days. Chondrogenesis was examined histologically, immunohistochemically, through biochemical assays and by RT-qPCR regarding the expression of chondrogenic marker genes.

**Results:**

Treatment of BMSCs with R57A led to a dose dependent induction of chondrogenesis in BMSCs. Biochemical assays also showed an elevated glycosaminoglycan (GAG) content and expression of chondrogenic marker genes in corresponding pellets. While treatment with R57A led to superior chondrogenic differentiation compared to treatment with the GDF-5 wild type and similar levels compared to incubation with TGF-ß1, levels of chondrogenic hypertrophy were lower after induction with R57A and the GDF-5 wild type.

**Conclusions:**

R57A is a stronger inducer of chondrogenesis in BMSCs than the GDF-5 wild type while leading to lower levels of chondrogenic hypertrophy in comparison with TGF-ß1.

## Background

Limited regenerative capacity of hyaline cartilage in combination with demographic changes have led to a sharp rise of patients suffering from osteoarthritis (OA) as well as the number of total joint arthroplasties [20]. Although patient satisfaction after primary hip or knee arthroplasty is high, joint replacements are limited by service life and revision, surgery remains complicated [17, 22, 50]. This has led to increasing interest in cell-based regenerative treatments for OA involving mesenchymal stromal cells (MSCs) [3]. MSCs can be isolated from various adult tissues. As defined by the International Society for Cellular Therapy (ISCT) MSCs express a characteristic set of surface antigens, are multipotent and adhere to plastic [16].

In order to optimize chondrogenic differentiation potential and limit unwanted hypertrophy during chondrogenesis of MSCs we and others investigated different combinations of scaffolds, growth factors and signalling pathways [23, 30]. Nonetheless, the ideal combination of growth factors has not yet been discovered [42]. Our earlier studies proved that various members of the transforming growth factor (TGF)-ß superfamily such as TGF-ß1, bone morphogenetic protein (BMP)-2 and BMP-4 promote and influence chondrogenic differentiation of MSCs in vitro [36, 41, 43, 44].

However, increasing cell hypertrophy characterized by the elevated expression and formation of collagen type X (COL X; encoded by COL10A1) remains one of the major obstacles observed during late chondrogenesis of MSCs in vitro. Chondrogenic hypertrophy signals the preliminary stage prior to cell apoptosis or mineralization of the extracellular matrix (ECM). This process naturally occurs during endochondral ossification (EO) in the growth plate leading to a replacement of hyaline cartilage with mineralized bone tissue but complicates cell-based methods of cartilage repair [4, 25, 42]. In accordance, in vivo data have shown that treatment of cartilage defects with MSCs and chondrogenic growth factors may lead to formation of osteophytes and tissue hypertrophy [10, 41]. Interestingly, earlier research has shown that transcription factors such as sex-determining region Y-type high-mobility-group-box (SOX) 9 (encoded by *SOX9*) not only promote chondrogenesis but also limit unwanted hypertrophy during chondrogenic differentiation of MSCs [48, 51].

Advanced understanding of different effects of growth factors on MSCs have led to extensive research regarding other growth factors influencing chondrogenesis and chondrogenic hypertrophy such as growth differentiation factor-5 (GDF-5) [8, 14, 35]. GDF-5 is a member of the TGF-ß superfamily and plays an important role during early bone and cartilage formation by increasing cell adhesion and therefore promoting prechondrogenic condensation [5]. Earlier research showed that GDF5 may regulate cartilage homeostasis by enhancing the production of matrix components in healthy chondrocytes and simultaneously limiting the activity of different proteases [7, 12]. Further, in vitro exposure of MSCs to GDF-5 led to an increased expression of chondrogenic and hypertrophy markers [1, 2, 9]. Just like BMP-2 and BMP-4 the GDF-5 wildtype binds to two variations of the type I BMP-receptor (BRI) called BRIA and BRIB [21]. Although the structure of GDF-5 is highly similar to that of BMP-2, the affinity of GDF-5 to BRIB is almost 12-fold higher than to BRIA [19]. Interestingly, this receptor affinity can be abrogated by exchanging Arginine 57 in GDF-5 with Alanine which leads to the generation of the mutant GDF-5 R57A (R57A) [19]. Previous studies have shown that while R57A mimics the receptor affinity to BRIA of BMP-2 it suppressed BMP-2-mediated expression of alkaline phosphatase (ALP; encoded by *ALP*) a marker gene for chondrogenic hypertrophy [19, 40, 44].

Therefore, the goal of our current in vitro study was to further examine possible differences regarding the effects of treatment with GDF-5 and the mutant R57A on chondrogenic differentiation and hypertrophy in bone marrow-derived MSCs (BMSCs) during pellet culture in vitro.

## Methods

### Generation and growth of GDF-5 and R57A

Plasmids harboring cDNAs which encode for the mature parts of either GDF5 or the mutein GDF5- R57A were both generated by standard cloning techniques and cloned into the expression plasmid RBSIIN25x/o as described earlier [31]. The resulting plasmids were then transformed into the E. coli strain BL21 (DE3). Protein expression was initiated in bacterial cultures by adding Isoropyl-thiogalactoside (IPTG) to a final concentration of 1 mM. After three hours of incubation at 37 °C cells were harvested by centrifugation and lyzed by sonication. The proteins were subsequently isolated from inclusion bodies, refolded and purified by cation exchange chromatography using SP-Sepharose as resin as described [31]. Purified proteins were afterwards dialyzed against water and stored at -80 °C until further use.

### Cultivation of BMSCs

Human BMSCs were isolated from 5 different donors aged from 33 to 65 years (mean age 53 years). All donors underwent total hip arthroplasty (THA) following informed consent and as approved by the institutional review board of the University of Würzburg as reported in our earlier studies [33, 44]. Bone marrow was harvested from the femoral head. Collected samples were spun, resuspended and seeded in plastic cell culture flasks (Greiner Bio-One GmbH, Frickenhausen, Germany). Harvested cells were cultivated in standard cell culture medium containing DMEM/Ham’s F12 supplemented, 10% fetal bovine serum (FBS) and 1% penicillin/streptomycin (PS) (all Life Technologies, Thermo Fischer Scientific, Dreieich, Germany). Culture medium changes were performed every 2 to 3 days (d). After reaching confluency cells were trypsinated, counted and placed in pellet cultures as subsequently described.

### Pellet cell culture

BMSCs were placed in pellet cultures as described in our previous studies [44]. To summarize BMSCs were resuspended in serum free DMEM supplemented with 1% ITS + Premix, 1 × 10^–7^ mol/l dexamethasone, 37.5 mg/ml Ascorbat-2-phosphate and 1 mM pyruvate (all Sigma, St Louis, MO, USA). Next 300 μl aliquots containing 3 × 10^5^ cells were placed in V-bottomed 96-well plates (Corning, Corning, NY, USA) to promote formation of small aggregates. Untreated controls were maintained as negative controls and cultured in the standard medium mentioned above. Other samples were incubated with 500 ng/ml or 1000 ng/ml of R57A or GDF-5. Lastly pellet cultures treated with 10 ng/ml recombinant TGF-ß1 (R&D Systems, Minneapolis, MN, USA) served as internal controls to compare effects regarding successful chondrogenesis and chondrogenic hypertrophy [45, 49, 51]. After cultivation at 37 °C, 5% CO_2_ pellet formation was observed within the first 24 h. Medium changes were performed every 2 to 3 d before pellets were harvested after a total of 21 d of chondrogenesis.

### Histology and immunohistochemistry

Pellets aggregates were dehydrated, embedded in paraffin and sectioned after being fixed in 4% paraformaldehyde (all Sigma) as described previously [44]. Alcian Blue (Sigma) stainings for detection of proteoglycans in pellet sections were performed as outlined in our earlier research [44]. Further, immunohistochemical stainings were performed using following compilation of antibodies and pre-digestion: collagen type II (COL II; encoded by COL2A1)-pepsin (1 mg/ml; Sigma)/monoclonal anti-COL II antibodies (Acris Antibodies GmbH, Hiddenhausen, Germany) and COL X—0.25% trypsin (Sigma)/polyclonal anti-COL X antibodies (Calbiochem, Bad Soden, Germany). Diaminobenzidine staining (DAB Kit; Sigma) following treatment with Advance™ HRP link and Advance™ HRP enzyme (Dako, Hamburg, Germany) was used to visualize immunohistochemical stainings. Sections were subsequently counterstained with hemalaun (Merck, Darmstadt, Germany). Non-immune IgGs (Sigma) replacing primary antibodies were used as negative controls. For detailed information regarding antibodies and immunohistochemistry please refer to our previous work [45, 49, 51].

### Biochemical assay

Biochemical assays were used to investigate cell proliferation, formation of glycosaminoglycans (GAG) and alkaline phosphatase (ALP) activity at 3, 7, 14 and 21 d as described earlier and following the respective user's manual [44]. Adenosine triphosphate (ATP) assays using the CellTiter-Glo® Luminescent Cell Viability Assay (Promega, Madison, WI, USA) were used to examine cell proliferation. After digestion with papain (1 μg/ml; Sigma) the content of GAG in harvested pellets was measured with the Blyscan™ Sulfated Glycosaminoglycan Assay (Biocolor Ltd, Newtownabbey, Northern Ireland) by reaction with 1,9-dimethylmethylene blue. The ALP activity was measured in an Enzyme-linked Immunosorbent Assay (ELISA) reader by the conversion of p-nitrophenol-phosphate to p-nitrophenol and inorganic phosphate via absorbance at 405 nm to determine the Quant-iT™ PicoGreen® kit (Invitrogen GmbH, Darmstadt, Germany).

### Isolation of RNA and RT-qPCR analysis

During chondrogenic differentiation RNA was extracted from BMSCs pellets at 3, 7, 14 and 21 d using Trizol reagent and purification steps involving DNAse (Invitrogen) treatment following the user's manual of the NucleoSpin RNA II kit (Macherey–Nagel GmbH, Düren, Germany). At days 3, 7, 14 and 21 RNA was extracted from the pellets. For the reverse transcription random hexamer primers (Table 1) and Bio-Script reverse transcriptase (Bioline GmbH, Luckenwalde, Germany) were used with 2 μg RNA from each group. Quantitative PCR (qPCR) was performed in triplicate with 1 μL cDNA, 10 μL KAPA SYBR FAST Universal 2 × qPCR Master Mix (peqlab Biotechnologie GmbH) and 1 μL of gene specific primers. The quantitative RT-PCR (RT-qPCR) was performed eventually with Opticon DANN Engine (MJ Research, Waltham, USA) using the following protocol: 95˚C for 3 min; 40 cycles: 95˚C for 15 s; 58˚C for 20 s; 72˚C for 30 s; the melting curve was analyzed after the last cycle. Calculation of the results was performed using the ΔΔ-CT Method. All sequences, annealing temperatures, cycle numbers and product sizes of forward and reverse primers used for COL2A1, SOX9, COL10A1 and ALP are listed in Table 1. As pointed out in our previous studies Elongation factor 1α (encoded by EEF1A) was used as the housekeeping gene [52].

**Table 1:** Primer details for quantitative RT-PCR. EEF1A, Elongation factor 1α; COL2A1, collagen type II alpha 1; COL10A1, collagen type X alpha 1; ALP, alkaline phosphatase; SOX9, sex-determining region Y-box 9

Gene	primer sequences (5'-3')	annealing temperature (°C)	product size (base pairs)	cycles	MgCl_2_
*Housekeeping gene for internal control*					
* EEF1A*	Sense: AGGTGATTATCCTGAACCATCC Antisense: AAAGGTGGATAGTCTGAGAAGC	54.0	234	25	1 x
*Chondrogenic marker genes*					
* COL2A1*	Sense: TTTCCCAGGTCAAGATGGTC Antisense: CTTCAGCACCTGTCCACCA	58.0	374	35	1x
* SOX9*	Sense: ATCTGAAGAAGGAGAGCGAG Antisense: TCAGAAGTCTCCAGAGCTTG	58.0	263	35	1x
*Chondrogenic hypertrophy marker genes*					
* COL10A1*	Sense: CCCTTTTTGCTGCTAGTATCC Antisense: CTGTTGTCCAGGTTTTCCTGGCAC	54.0	468	25	1x
* ALP*	Sense: TGGAGCTTCAGAAGCTCAACACCA Antisense: ATCTCGTTGTCTGAGTACCAGTCC	51.0	454	35	1x

### Statistical analysis

All data derived from experiments involving ATP assays, GAG assays, DNA assays, ALP assays or RT-PCR were performed using BMSCs derived from five different donors (*n* = 5) and expressed as mean values ± standard deviation. Each experiment was performed in triplicate or quadruplicate (*n* = 3 to 4). Data was checked for normal distribution using the Kolmogorov–Smirnov and Shapiro–Wilk test. Non-parametric testing was performed in case of not normally distributed data. For evaluation of statistically significant expression rates in the ATP assays, GAG assays and DNA assays for the R57A, GDF-5 und TGF-ß incubation medium, respectively, the Kruskal–Wallis-Test including a post-hoc Dunn-Bonferroni-Test was used. Differences in the relative expression level of chondrogenic and hypertrophic marker genes were also determined using the Kruskal–Wallis-Test and statistically significant differences between the R57A, GDF-5 and TGF-ß incubation medium were evaluated using the post-hoc Dunn-Bonferroni Test. *P*-values < 0.05 were considered statistically significant.

## Results

### Histological and immunohistochemical analysis of chondrogenic differentiation

**Fig. 1:**
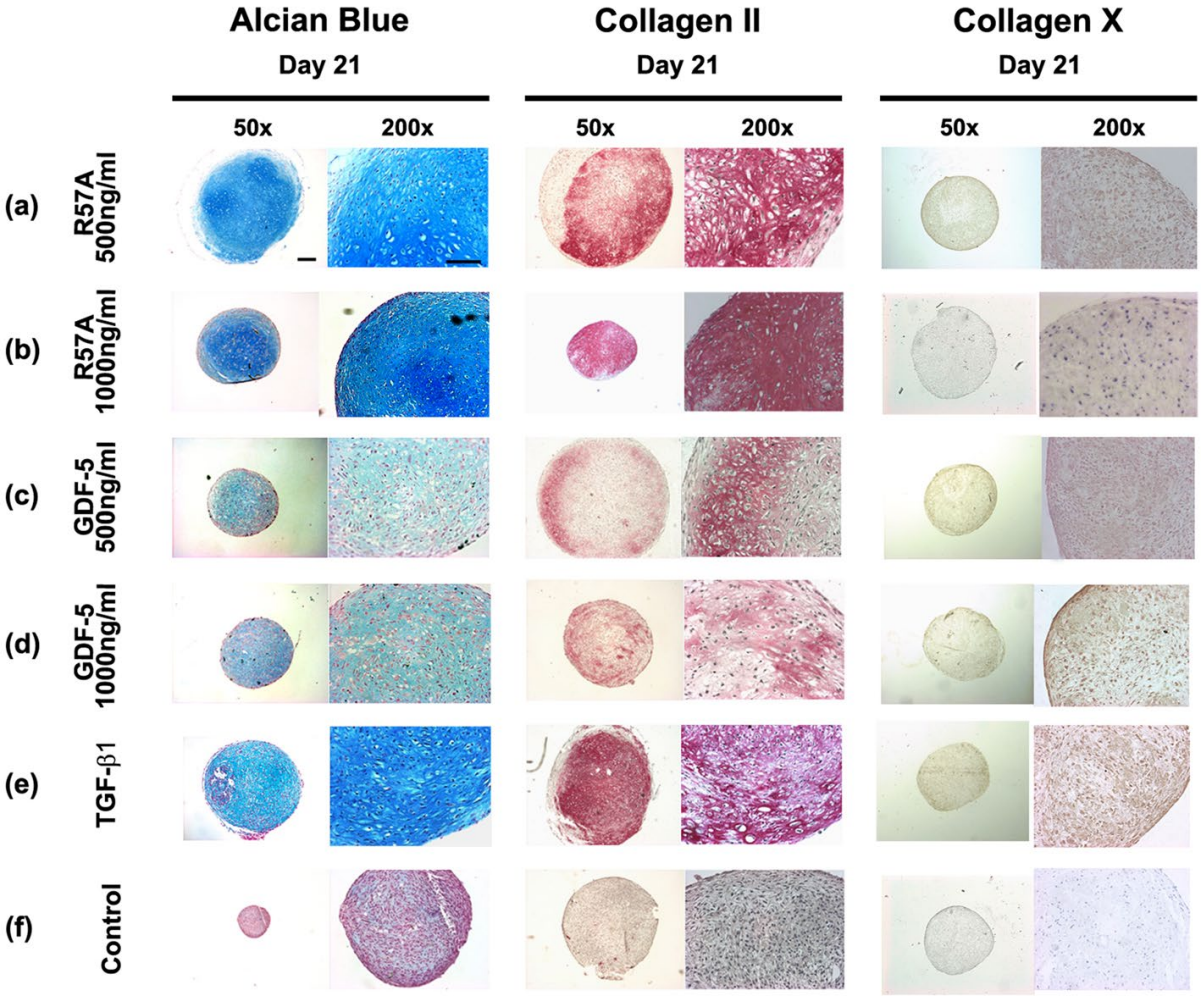
Histological and immunohistochemical analysis of chondrogenic differentiation and hypertrophic differentiation in mesenchymal stromal cells after incubation with R57A, GDF-5 and TGF-ß1 21 days in pellet culture. Mesenchymal stromal cells (MSCs) were incubated with 500 ng/ml (**a**) and 1000 ng/ml (**b**) of the mutant R57A, 500 ng/ml (**c**) and 1000 ng/ml (**d**) of growth and differentiation factor 5 (GDF-5) or transforming growth factor beta (TGF-β)1 (**e**) in pellet cultures for 21 days. Untreated cultures treated with standard cell culture medium were maintained as negative controls (**f**). Clear signs of chondrogenesis as judged by histological Alcian Blue stainings and immunohistochemical stainings of Collagen type II (Collagen II) were observed in all pellet cultures treated with R57A, GDF-5 and TGF-ß1 (Alcian Blue, Collagen II, **a-e**) while internal controls showed no positive staining (Alcian Blue, Collagen II, **f**). All cultures showed slightly positive immunohistochemical stainings of the hypertrophy marker collagen type X (Collagen X, **a-e**). Representative samples were captured at low (50x; black bar = 100 μm) and high (200x; black bar = 50 μm) magnification. GDF-5, growth and differentiation factor 5; MSCs, mesenchymal stromal cells; TGF-ß transforming growth factor beta

After 21 d all differentiated pellets showed clear signs of chondrogenic differentiation as judged by Alcian Blue stainings after treatment with R57A (500 ng/ml and 1000 ng/ml) and GDF-5 (500 ng/ml and 1000 ng/ml) or incubation with TGF-ß1 (Fig. 1, Alcian Blue, a-e). Similar results were observed for immunohistochemical stainings of COL II (Fig. 1, Collagen II, a-e). Intensity of Alcian blue stainings and immunohistochemical stainings of COL II increased in dose dependent manner after incubation of BMSCs with R57A (Fig. 1, Alcian Blue/Collagen II, a-b). Alcian Blue staining intensity and intensity of immunohistochemical stainings of COL II was highest after treatment with 1000 ng/ml of R57A (Fig. 1, Alcian Blue/Collagen II, b). Staining intensity was lower in differentiated cultures treated with 500 ng/ml of R57A and TGF-ß1 in comparison to other differentiated cultures treated with 1000 ng/ml of R57A and GDF-5 (500 ng/ml and 1000 ng/ml) (R57A (Fig. 1, Alcian Blue/Collagen II, a, e). Staining intensity was lowest in differentiated pellet cultures treated with GDF-5 independent of concentrations used (Fig. 1, Alcian Blue/ Collagen II, c-d).

Regarding the phenotype of pellet sections respective pellets treated with R57A and TGF-ß1 showed more chondrone-like structures (Fig. 1, Alcian Blue, a-b, e). Pellets treated with R57A also showed more homogenic stainings of COL II following chondrogenic differentiation (Fig. 1, Collagen II, a-b).

Controls showed negative Alcian Blue stainings and negative immunohistochemical stainings for COL II (Fig. 1, Alcian Blue/Collagen II, f).

In addition, controls and pellets which were treated with 10 ng/ml TGF-ß1as well as GDF-5 independent of the concentrations used showed clearly positive immunohistochemically stainings for the marker of chondrogenic hypertrophy COL X (Fig. 1, Collagen X, c-e). In comparison, staining intensity regarding immunohistochemically staining of COL X seemed lower in pellets which were treated with 500 ng/ml and 1000 ng/ml of R57A (Fig. 1, Collagen X, a-b).

### Biochemical analysis of cell proliferation, GAG content and ALP activity

**Fig. 2:**
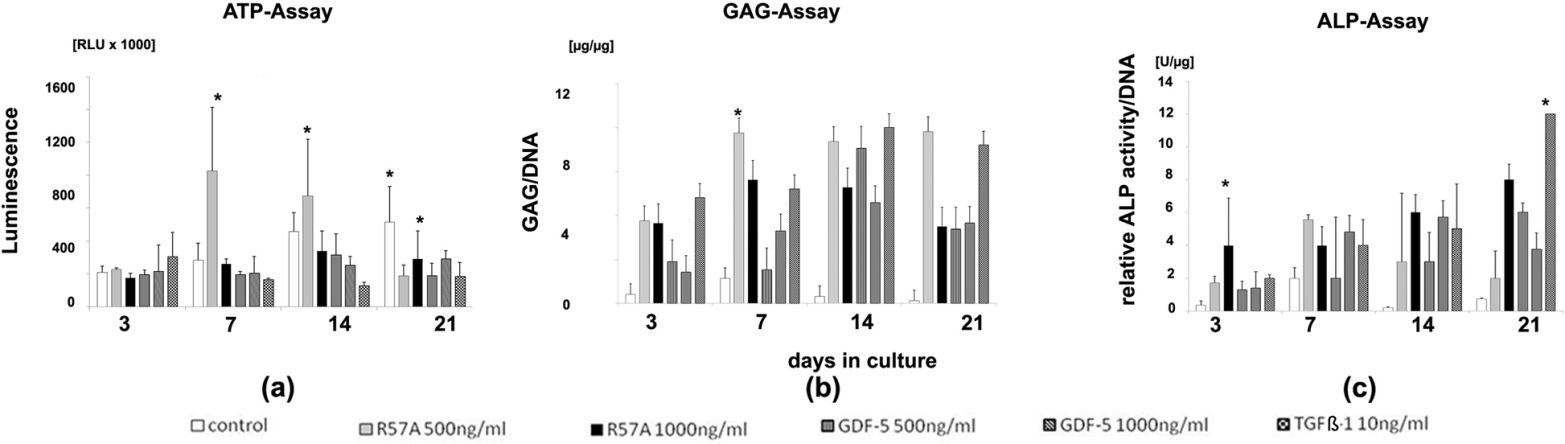
Assay of cell proliferation ATP activity, GAG content and ALP activity after 21 days of chondrogenic differentiation in pellet cultures derived from five different donors. Pellet cell cultures containing mesenchymal stromal cells (MSCs) were incubated in chondrogenic differentiation medium containing 500 ng/ml and 1000 ng/ml of the mutant R57A, 500 ng/ml and 1000 ng/ml of growth and differentiation factor 5 (GDF-5) or transforming growth factor beta (TGF-β)1 for 21 days to induce chondrogenesis. Untreated cultures treated with standard cell culture medium were maintained as negative controls. Adenosine Triphosphate (ATP)-Assays (**a**) were performed to examine cell proliferation after 3, 7, 14 and 21 days. Glycosaminoglycan (GAG)-Assays (**b**) were conducted to examine the quantitative synthesis of the chondrogenic marker protein GAG in respective pellets after 3, 7, 14 and 21 days. In addition, alkaline phosphatase (ALP)-Assays (**c**) were performed to evaluate relative ALP-activity after 3, 7, 14 and 21 days. Asterixis (*) represent significant differences (*P* < 0.05) compared to negative control samples at this particular time. The respective group of pictured bars refer to MSCs treated as controls, with R57A 500 ng/ml, with R57A 1000 ng/ml, with GDF-5 500 ng/ml, with GDF-5 1000 ng/ml and TGF-ß1 10 ng/ml (from left to right). ATP, Adenosine Triphosphate; ALP, alkaline phosphatase; GAG, Glycosaminoglycan; GDF-5, growth and differentiation factor 5; MSCs, mesenchymal stromal cells; TGF-ß, transforming growth factor beta

ATP assays showed a steady increase of cell proliferation in control cultures over 21 d (Fig. 2, a, control). Cell proliferation remained mostly constant in differentiated pellet cultures during the same time span (Fig. 2, a). Interestingly cell proliferation in pellets increased after 7 d before declining again until d 21 after treatment with 500 ng/ml R57A (Fig. 2, a, R57A 500 ng/ml). After 7 d of chondrogenesis cell proliferation was significantly higher in cultures treated with 500 ng/ml R57A (Fig. 2, a, 7, R57A 500 ng/ml) and after 14 d cell proliferation was significantly lower in cultures treated with 10 ng/ml TGF-ß1 (Fig. 2, a, 14, TGF-ß1 10 ng/ml) in comparison to other differentiated cultures and negative controls. Besides these findings cell proliferation did not differ significantly at 3, 7, 14 or 21 d after treatment R57A, TGF-ß1 or GDF-5 independent of concentrations used (Fig. 2, a).

Quantitative examination of GAG synthesis showed little increase in control cultures on day 7 but did not differ significantly in comparison to points of evaluation over the course of 21 d (Fig. 2, b, control). Pellet cultures treated with 500 or 1000 ng/ml of R57A, 1000 ng/ml of GDF-5 and TGF-ß1 showed an increase of GAG content in comparison to control cultures after 7 d of chondrogenesis (Fig. 2, b, 7). A similar trend was observed after 14 d of treatment of pellet cultures with 500 ng/ml of GDF-5 (Fig. 2, b, 14). GAG synthesis in differentiated pellet cultures peaked after 14 d (Fig. 2, b, 14) besides from pellet cultures treated with 500 ng/ml R57A which reached highest values after 21 d of chondrogenic differentiation (Fig. 2, b, 21 days in culture). After 7 d of chondrogenesis GAG synthesis was significantly higher in cultures treated with 500 ng/ml R57A (Fig. 2, b, 7, R57A 500 ng/ml) and after 21 d GAG synthesis was significantly higher in cultures treated with 500 ng/ml R57A (Fig. 2, b, 21, R57A 500 ng/ml) and 10 ng/ml TGF-ß1 (Fig. 2, b, 21, TGF-ß1 10 ng/ml) in comparison to other differentiated cultures and negative controls.

Further relative ALP activity was elevated in all chondrogenic differentiated pellet cultures in comparison to control cultures during 21 d of chondrogenesis (Fig. 2, c). After 14 and 21 d of chondrogenesis ALP activity was increased in all chondrogenic differentiated pellets in comparison to control cultures (Fig. 2, c, 14—21). Interestingly, relative ALP activity decreased between 7 and 21 d after treatment with 500 ng/ml of R57A (Fig. 2, c, 7—21, R57A 500 ng/ml). Finally, relative ALP activity was significantly higher in pellets treated with TGF-ß1 (Fig. 2, c, 21, TGF-ß1 10 ng/ml) and 1000 ng/ml of R57a (Fig. 2, c, 21, R57A 1000 ng/ml) after 21 d of chondrogenesis.

### RT-qPCR analysis of the expression of chondrogenic and hypertrophic marker genes

**Fig. 3:**
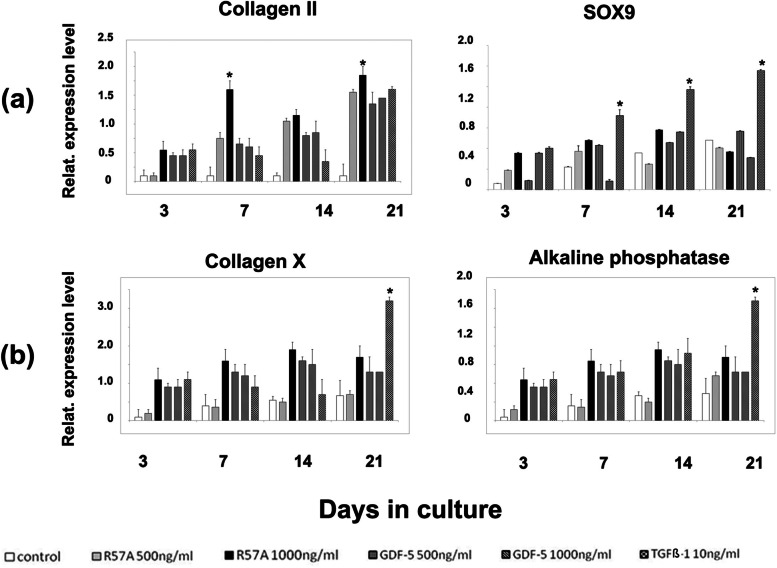
Mean changes in the relative expression of chondrogenic and hypertrophic marker genes ± standard deviation as measured by RT-qPCR in mesenchymal progenitor cells at the end of the chondrogenic differentiation period. Mesenchymal stromal cells (MSCs) derived from bone marrow of five different patients were incubated with chondrogenic differentiation medium containing 500 ng/ml and 1000 ng/ml of the mutant R57A, 500 ng/ml and 1000 ng/ml of growth and differentiation factor 5 (GDF-5) or transforming growth factor beta (TGF-β)1 for 21 days for 21 days. Untreated cultures treated with standard cell culture medium were maintained as negative controls. The mean changes in the relative expression of the chondrogenic (**a**) marker genes—collagen type II (Collagen II) and sex-determining region Y-type high-mobility-group-box (SOX) 9—as well as the hypertrophic (**b**) marker genes—collagen type X (Collagen X) and alkaline phosphatase (ALP)—are pictured, respectively. Error bars represent the range of changes in the mean expression of specific marker genes in differentiated cell cultures from which the standard deviations were calculated. Elongation factor 1α (EEF1α) was used as the housekeeping gene and for internal controls. Primer details are illustrated in Table 1. Asterixis (*) represent significant differences (*P* < 0.05) compared to negative control samples at this particular time. The respective group of pictured bars refer to MSCs treated as controls, with R57A 500 ng/ml, with R57A 1000 ng/ml, with GDF-5 500 ng/ml, with GDF-5 1000 ng/ml and TGF-ß1 10 ng/ml (from left to right). RT-PCR was performed using BMSCs derived from five different donors (*n* = 5) and each experiment was performed in triplicate or quadruplicate (*n* = 3 to 4). d, days; GDF-5, growth and differentiation factor 5; TGF-ß, transforming growth factor beta

To compare effects of treatment of BMSCs with R57A, GDF-5 and TGF-ß1 on chondrogenesis and possible hypertrophic differentiation we examined the relative expression of respective marker genes after 3, 7, 14 and 21 d (Fig. 3). In this context COL2A1 and COL10A1 refer to the genes coding the respective COL II and COL X alpha 1 chains.

Control cultures expressed low levels of chondrogenic marker genes COL2A1 and SOX9 over the course of 21 d of chondrogenesis. In contrast, the expression of all chondrogenic marker genes were elevated in all chondrogenic differentiated cultures treated with R57A, GDF-5 or TGF-ß1 after 14 and 21 d of chondrogenesis in comparison to control cultures (Fig. 3, Collagen II/ SOX9, 14—21). Expression of COL2A1 was significantly higher in cultures treated with 1000 ng/ml R57A in comparison to control cultures after 7 and 21 d of chondrogenesis (Fig. 3, Collagen II, 7, R57A 1000 ng/ml). Expression of SOX9 was significantly higher in pellets treated with 10 ng/ml TGF-ß1 after 7, 14 and 21 d of chondrogenesis in comparison to control cultures (Fig. 3, SOX9, 7—21, TGF-ß1 10 ng/ml).

Further, 21 d of chondrogenesis led to non-significant elevation of relative gene expression levels of hypertrophy marker gene COL10A1 in all chondrogenic differentiated cultures in comparison to control cultures over the course of 21 d (Fig. 3, Collagen X, 3—21).

Gene expression of ALP showed slight upregulation during 21 d of pellet culture (Fig. 3, Alkaline phosphatase, 3—21). Interestingly, at 14 and 21 d expression of ALP increased in all chondrogenic differentiated cultures (Fig. 3, Alkaline phosphatase, 14—21). However, gene expression of ALP remained lowest in pellets treated with 500 ng/ml R57A (Fig. 3, Alkaline phosphatase, 21). However, the expression of COL10A1 and ALP was significantly higher in pellet cultures treated with TGF-ß1 after 21 d of chondrogenesis (Fig. 3, Collagen X/Alkaline phosphatase, 21, TGF-ß1 10 ng/ml) in comparison to differentiated cultures or negative controls.

## Discussion

MSCs have emerged as a promising cell source for the regenerative treatment of articular cartilage defects using tissue engineering. However, terminal hypertrophic differentiation of MSCs as seen during endochondral ossification remains a major obstacle in cell-based cartilage repair [11, 26, 42].

We and others have previously shown that the delivery of different transcription and growth factors to MSCs can enhance chondrogenic differentiation while simultaneously promoting or mitigating hypertrophic differentiation [36, 43, 44, 52]. While different members of the TGF-ß superfamily led to progression towards chondrogenic hypertrophy gene transfer of *SOX9* showed a decreased trend towards chondrogenic hypertrophy during chondrogenic differentiation [51]. In our current study we examined the effects of different doses (500 ng/ml and 1000 ng/ml) of GDF-5, the mutant R57A as well as TGF-ß1 on chondrogenic differentiation of human BMSCs.

Our present in vitro study showed that treatment of BMSCs with different doses of GDF-5, R57A and TGF-ß1 led to successful chondrogenic differentiation as shown by Alcian Blue stainings, immunohistochemical stainings of COL II and elevated relative expression of chondrogenic marker genes. Intensity of Alcian Blue and COL II stainings seemed to be stronger in pellet cultures after treatment with R57A and TGF-ß1 when compared to pellets treated with GDF-5 independent of the concentrations used. After 21 d of chondrogenesis GAG content increased in all differentiated pellet cultures in comparison to control cultures. After 21 d of chondrogenesis GAG content was significantly higher in pellet cultures treated with 1000 ng/ml R57A and 10 ng/ml TGF-ß1 in comparison to other differentiated cultures and negative controls. In addition, chondrogenic differentiation of BMSCs led to increased relative expression of chondrogenic marker genes *COL2A1* and *SOX9* in comparison to control cultures. After 7 and 21 d of chondrogenesis the expression levels of chondrogenic marker genes were significantly higher in pellets treated with 1000 ng/ml R57A and 10 ng/ml TGF-ß1. In addition, we observed intense immunohistochemical stainings of the hypertrophy marker COL X after treatment with GDF-5 and TGF-ß1. After 21 d of chondrogenesis all differentiated pellet cultures showed an increased expression of *ALP* in comparison to control cultures*.* After 21 d of chondrogenic differentiation pellet cultures treated with 10 ng/ml TGF-ß1 showed a significant increase in the expression levels for both *ALP* and *COL10A1*. In addition, 21 d of chondrogenesis in pellet culture also led to a significantly increased ALP activity in cultures treated with 1000 ng/ml R57A and 10 ng/ml TGF-ß1. This is in line with our previous studies in which incubation of pellet cultures with TGF-ß1 led to clear chondrogenic differentiation accompanied by stronger hypertrophic and osteogenic de-differentiation in comparison to other prochondrogenic transcription or growth factors [51]. While the incubation of BMSC with the growth factors R57A, GDF-5 and TGF-ß1 for 21 d in pellet cultures in vitro led to successful chondrogenic differentiation, TGF-ß1 and higher concentrations of R57A may promote hypertrophic and osteogenic differentiation. Differences regarding the immunohistochemical staining of COL X and RT-qPCR results may be due to phasal upregulation resulting in delayed protein formation and expression of respective marker genes [49].

Earlier research by Coleman et al. found that incubation of BMSCs with GDF-5 led to significantly enhanced chondrogenesis as shown by increased production of COL II and GAG [9]. Despite these findings incubation with GDF-5 simultaneously led to increased SMAD phosphorylation and elevated relative expression of hypertrophic marker genes such as *COL10A1* and *ALP *[9]. Previous studies linked SMAD phosphorylation to increased expression of hypertrophic marker genes and mineralization of the ECM [15]. Similar results were found after incubation of embryonic stem cells (ESCs), embryonic chick mesenchymal cultures or other MSC-subpopulations with GDF-5 in vitro [8, 34].

Further Fang et al. showed that adenoviral gene transfer of GDF-5 to MSCs derived from adipose tissue led to enhanced chondrogenic differentiation as shown by increased production of GAG and elevated expression of chondrogenic marker genes [14]. In contrast to the findings in this study Fang et al. showed that adenoviral gene transfer of GDF-5 led to superior effects on chondrogenesis in MSCs compared to incubation with TGF-ß1 [14]. Similar to our present study Xiaowen et al. confirmed pro-chondrogenic effects of GDF-5 on fetal MSCs but found GDF-5 to be less stimulatory than TGF-ß1 [2]. Interestingly, combined treatment with GDF-5 and TGF-ß1 had synergistic effects on chondrogenesis [2].

However, in line with our results hypertrophic differentiation and increased expression of hypertrophic or osteogenic marker genes was observed in most of the studies which examined the effects of GDF-5 on chondrogenesis in multiple cell types [2, 8, 9, 14]. In line with these results adenoviral gene transfer leading to overexpression of GDF-5 in MSCs led to osteogenic differentiation in vitro and in vivo [6]. Interestingly, incubations of MSCs with GDF-5 in hypoxic conditions led to increased chondrogenic differentiation as well as decreased hypertrophic differentiation as shown by expression of COL X in comparison to normoxic conditions [47].

GDF-5 is also known as cartilage-derived morphogenetic protein-1 (CDMP-1) or BMP-14 and is closely related to other members of the TGF-ß superfamily such as BMP-2 [19, 39]. Earlier research revealed that GDF-5 acts by binding to the transmembrane serine/threonine kinase receptors BRIA, BRIB and BRII [19]. Binding to these receptors activates downstream SMAD dependent pathways through phosphorylation [9, 46]. Activation of SMAD pathways is closely associated with cell proliferation, cell differentiation and changes of the gene expression of chondrogenic marker genes such as *COL2A1* or aggrecan (encoded by *ACAN*) [29, 46]. Chondrogenesis involves the condensation, differentiation and maturation of MSCs. During this process GDF-5 has been shown to promote cell adherence similar to pellet cell-culture supporting enhanced prechondrogenic cell condensation [8, 24]. Interestingly, single-nucleotide polymorphisms (SNPs) of the *GDF-5* gene are associated with susceptibility to OA [13, 28]. In addition, GDF-5 expression has shown to be upregulated in chondrocytes derived from articular cartilage in patients suffering from OA [37].

During embryological limb development condensed cells differentiate into chondrocytes which later become hypertrophic before calcifying, undergoing apoptosis and being replaced by bone tissue [8]. While GDF-5 is believed to play an important role in homeostasis of adult hyaline cartilage, research has shown that GDF-5 also promotes cell condensation and hypertrophic differentiation during limb development and chondrogenic differentiation of MSCs in vitro [6, 9, 46]. In accordance, research has linked mutations of GDF-5 to multiple cartilage and joint disorders [8, 18]. Out of all BRI receptor variations GDF-5 naturally shows a tenfold greater binding affinity to BRIB in comparison to BRIA. In contrast, BMP2 possesses similar binding affinity to BRIA and BRIB [19, 32]. We and others have shown that incubation of MSCs with BMP2 or genetic transfer of BMP2 to MSCs has shown to significantly enhance chondrogenesis in vitro and in vivo [38]. However, BMP2 has also shown to promote osteogenesis in MSCs via SMAD-dependent pathways as shown by increased expression of osteogenic marker genes and formation of specific matrix molecules [38]. Concordant with these findings BMP2 enhanced unwanted hypertrophic differentiation during chondrogenesis of MSCs in various earlier studies [41, 42, 52].

The mutant R57A is formed by exchanging Arginine 57 with Alanine. Nickel et al. pointed out that this exchange of amino acids in R57A led to a higher receptor affinity towards BRIA in comparison to GDF5 making R57A a BMP2-mimic [19, 32]. Previous research pointed out that GDF-5-mutants may affect osteogenesis and chondrogenesis in MSCs differently than the GDF-5-wildtype [27]. Klammert et al. showed that incubation of different cell lines with R57A or GDF-5 led to reduced overall ALP activity and dose-dependent inhibition of BMP2-mediated ALP activity [19]. Consistent with differences in the receptor affinity towards BRIA effects were greater when using R57A [19]. In addition, BMP2 led to endochondral, heterotopic ossification upon implantation in rat muscles while this was not the case for R57A and GDF-5 [19]. Coimplantation of BMP2 with GDF-5 or R57A led to reduced heterotopic ossification pointing out their assumed role as context-dependent BMP2-antagonists [19]. In our current study we found no differences regarding the effects of R57A and GDF-5 on hypertrophic differentiation in MSCs independent of the concentrations used.

In summary, our present in vitro study showed that GDF-5, R57A and TGF-ß1 all induce chondrogenic differentiation in pellet cultures of BMSCs. Although chondrogenic differentiation may be superior when using 500 ng/ml and 1000 ng/ml R57A as well as 10 ng/ml TGF-ß1 in comparison to GDF-5 as indicated by histological and immunohistochemical stainings, incubation with 1000 ng/ml R57A and 10 ng/ml TGF-ß1 also led to a significant increase in gene expression of hypertrophic marker genes and ALP activity. However, one major weakness to our current study is the missing quantitative analysis of histological stainings, meaning examination and comparison of chondrogenesis based on histological data is only a rough analysis based on the visual judgement of the researcher. Further in vitro and in vivo studies are necessary to further examine the pro-chondrogenic and hypertrophic potential of the mutant R57A in tissue engineering (TE) and therapeutically relevant differences compared to recombinant GDF-5.

## Conclusion

The high affinity variant R57A of the growth factor GDF-5 is a strong inducer of chondrogenesis in BMSC-pellet cultures. However, potential risk of ossification and hypertrophy during chondrogenic differentiation has to be considered. Further research has to focus on identifying appropriate biomaterials for the delivery of these factors to investigate the in vivo capability for TE based approaches in cartilage repair.

## Data Availability

The datasets used and analyzed during the current study are available from the corresponding author on reasonable request.

## Abbreviations

ALP (encoded by ALP): Alkaline phosphatase
BMSCs: Bone marrow-derived mesenchymal stromal cells
BMP: Bone Morphogenetic Protein
BR: BMP receptor
CDMP-1: Cartilage-derived morphogenetic protein-1
cDNA: Complementary DNA
COL II: Collagen type II
COL X: Collagen type X
COL2A1: collagen type II alpha 1
COL10A1: collagen type X alpha 1
d: Days
ECM: Extracellular matrix
EEF1A: Elongation factor 1α
EO: Endochondral ossification
FBS: Fetal bovine serum
GDF-5: Growth and differentiation factor 5
GDF-5 R57A: R57A
MSCs: Mesenchymal stromal cells
ISCT: International Society for Cellular Therapy
PBS: Phosphate buffered saline
PS: Penicillin/streptomycin
RT-qPCR: Quantitative reverse-transcriptase polymerase chain reaction
SMAD: Homologues of the Drosophila protein, mothers against decapentaplegic (Mad) and the Caenorhabditis elegans protein Sma
SOX9, Sox-9: Sex-determining region Y-box 9
TE: Tissue engineering
THA: Total hip arthroplasty
